# Translocation and Dissemination of Gut Bacteria after Severe Traumatic Brain Injury

**DOI:** 10.3390/microorganisms10102082

**Published:** 2022-10-21

**Authors:** Weijian Yang, Qiang Yuan, Zhiqi Li, Zhuoying Du, Gang Wu, Jian Yu, Jin Hu

**Affiliations:** 1 Department of Neurosurgery, Huashan Hospital, Shanghai Medical College, Fudan University, Shanghai 200040, China; 2 National Center for Neurological Disorders, Shanghai 200040, China; 3 Shanghai Key Laboratory of Brain Function Restoration and Neural Regeneration, Shanghai 200040, China; 4 Neurosurgical Institute of Fudan University, Shanghai 200040, China; 5 Shanghai Clinical Medical Center of Neurosurgery, Shanghai 200040, China

**Keywords:** traumatic brain injury, lung infection, bacterial translocation, Paneth cells, antimicrobial peptides

## Abstract

*Enterobacteriaceae* are often found in the lungs of patients with severe Traumatic Brain Injury (sTBI). However, it is unknown whether these bacteria come from the gut microbiota. To investigate this hypothesis, the mice model of sTBI was used in this study. After sTBI, Chao1 and Simpson index peaking at 7 d in the lungs (*p* < 0.05). The relative abundance of *Acinetobacter* in the lungs increased to 16.26% at 7 d after sTBI. The chao1 index of gut microbiota increased after sTBI and peaked at 7 d (*p* < 0.05). Three hours after sTBI, the conditional pathogens such as *Lachnoclostridium*, *Acinetobacter*, *Bacteroides* and *Streptococcus* grew significantly. At 7 d and 14 d, the histology scores in the sTBI group were significantly higher than the control group (*p* < 0.05). The myeloperoxidase (MPO) activity increased at all-time points after sTBI and peaked at 7 d (*p* < 0.05). The LBP and sCD14 peaking 7 d after sTBI (*p* < 0.05). The Zonulin increased significantly at 3 d after sTBI and maintained the high level (*p* < 0.05). SourceTracker identified that the lung tissue microbiota reflects 49.69% gut source at 7 d after sTBI. In the small intestine, sTBI induced gastrointestinal dysfunction with increased apoptosis and decreasing antimicrobial peptides. There was a negative correlation between gut conditional pathogens and the expression level of antimicrobial peptides in Paneth cells. Our data indicate that gut bacteria translocated to the lungs after sTBI, and Paneth cells may regulate gut microbiota stability and translocation.

## 1. Introduction

Traumatic brain injury (TBI) is one of the most fatal and disabling health problems in the world [1]. TBI is not limited to the central nervous system (CNS) but has a wide range of systemic effects, altering the biology and function of lung, intestine and gut microbiota. Gut microbiota comprises billions of microorganisms in the human gastrointestinal tract and exerts numerous physiological functions. Gut microbiota dysbiosis represents a critical factor in the pathogenesis of many local and systemic diseases [2]. It is reported that TBI results in the destruction of gut mucosal integrity, and the leaky gut may allow bacterial components to enter circulation [3]. In patients with severe TBI (sTBI), bacterial infection is a major risk factor for poor prognosis [4]. It was reported that the incidence of lung infection following TBI might be as high as 50–60%, with infection-related mortality as high as 30% [5]. Recent studies have shown that the lung microbiota of patients with sepsis, acute respiratory distress syndrome and stroke are often found to be rich in gut-related bacteria, primarily *Bacteroides* and *Enterobacteriaceae* [6,7,8,9]. Therefore, we proposed that there might be gut bacterial translocation to the lungs after sTBI.

Gastrointestinal dysfunction is a common complication in patients with brain injury [10]. Following brain injury, intestinal epithelial cell dysfunction, apoptosis or mucosal atrophy may occur [11]. The principal components of the intestinal niche are Paneth cells, which play important roles in intestinal stem cell homeostasis, development of the microbiome, and host defense against intestinal pathogens [12]. Paneth cells are important because they synthesize and secrete antimicrobial peptides in the small intestine, and their dysfunction may increase susceptibility to infection [13]. Paneth cells may play an important role in maintaining the homeostasis balance between normal microbiota, infectious pathogens and the human body, as well as regulating the qualitative composition and quantity of intestinal microorganisms [14]. Impairment of the number or function of Paneth cells is associated with a reduction in the clearance of bacterial pathogens. Although the complex relationship between gut and lung microbiota is not fully understood, there is growing evidence that intestinal-pulmonary axis disorder may play a key role in the pathogenesis of pulmonary complications in critically ill patients [15].

Here, we sought to determine the effects of sTBI on the stability of the gut and lung microbiota as well as the impact on Paneth cells and bacterial translocation in sTBI mice. We hypothesized that gut bacteria translocated to the lungs after sTBI and Paneth cells might regulate gut microbiota stability and translocation.

## 2. Materials and Methods

### 2.1. Mice and Modeling

#### 2.1.1. Mice and Housing

Male 6–8-week-old specific pathogen-free (SPF) C57BL/6 mice were purchased from Shanghai Lingchang Biotechnology Co., Ltd. (Shanghai, China) and housed under SPF conditions. The mice were acclimated for one week under conditions controlled by temperature (23 ± 2 °C) and humidity (50–60%), maintaining 12 h/12 h light/dark cycles and allowing mice to harvest energy freely. Sterilized drinking water was supplied timely with drinking bottles, which were washed every day. Sterilized cages and pads were changed every three days. Sixty-six mice were randomly distributed into the control group, sham group (five-time points), and sTBI group (five-time points). There were six mice in each group or time point. Time points were taken at 3 h, 1 d, 3 d, 7 d and 14 d after modeling. This study was approved by the ethics committee of Huashan Hospital, Affiliated with Fudan University (Ethics Number: 2018 Huashan Hospital JS-045), and registered in the Department of animal experiments to ensure the accuracy and repeatability of the experimental study. All experimental procedures and animal welfare standards strictly followed the guidelines of the Animal Experimental Research Report [16].

#### 2.1.2. Animal Model

In the sTBI group, mice were anesthetized by intraperitoneal injection of 0.6% sodium pentobarbital at a dose of 80 mg/kg. The bone window was located 1 mm behind the coronal suture and 3 mm on the left side of the sagittal suture. The bone window was drilled with a 4 mm diameter dental drill to expose the complete dura. The sTBI model was established by the Pinpoint PCI 3000 system (Hatteras Instruments Inc., Cary, NC, USA). The modified parameters were set as follows [17]: the diameter of the striking head was 3 mm, the depth was 3 mm, the strike speed was 1.5 m/s, and the strike time was 100 ms. The mice in the sham group received anesthesia and craniotomy. The mice in the control group were anesthetized only.

#### 2.1.3. Sample Collection

The mice were placed in a sterile mouse cage lined with sterile filter paper, and stool samples were collected using sterile tweezers immediately after defecation. The mice were placed individually in a sterile cage during fecal collection. Fresh mouse stool samples were collected and immediately stored at −80 °C. At the end of the experiment, mice were anesthetized with sodium pentobarbital and euthanized by exsanguination. The thorax was immediately opened, and the lungs were removed under sterile conditions. Subsequently, the right lung was stored at −80 °C until sequencing. The left lung was prepared for histochemical protein examination of proposed endpoints. The ilea were collected and sliced into two sections. One of these was stored in liquid nitrogen until western blot analysis and *real-time polymerase chain reaction* (RT-PCR). The other was fixed in 4% (*v*/*v*) paraformaldehyde and used in histological and immunohistochemical (IHC) analyses.

### 2.2. Protein and Messenger Ribonucleic Acid (mRNA) Detection

#### 2.2.1. Cleaved Caspase-3 and Lysozyme Assay by Western Blot

The expression levels of cleaved caspase-3 and lysozyme in small intestinal tissue were detected by western blot analysis. Pre-stained Protein Ladder (26616) was purchased from Thermo, Waltham, MA, USA. Immobilon Western HRP Substrate (WBKLS0500) and Hydrophobic PVDF Transfer Membrane (IPVH00010) were purchased from Millipore, Burlington, MA, USA. Anti-lysozyme antibody (ab108508) was purchased from Abcam, Cambridge, UK. β-actin (4970) and Cleaved Caspase-3 (9664) were purchased from CST, Danvers, MA, USA. The Western Blot was analyzed by Image J (1.8.0_172), USA. The data were analyzed by the genescloud tools, a free online platform for data analysis (https://www.genescloud.cn, accessed on 29 July 2020).

#### 2.2.2. mLyz1 and mDefa6 Assay by Quantitative RT-PCR

Primers were used for RT-PCR analysis as previously described [13]. Primer information: mLyz1-F, GGAATGGATGGCTACCGTGG (5’ to 3′); mLyz1-R, CATGCCACCCATGCTCGAAT (5′ to 3′); mDefa6-F, CCTTCCAGGTCCAGGCTGAT (5’ to 3’), mDefa6-R, TGAGAAGTGGTCATCAGGCAC (5′ to 3′); GAPDH-F, TGTTTCCTCGTCCCGTAGA (5′ to 3′), GAPDH-R, ATCTCCACTTTGCCACTGC. The PCR was performed in two steps of 5 min-pre-heat at 95 °C and 30 s at 60 °C extensions for 40 cycles after a 15 s at 95 °C denature. The amplification curve of the PCR product was obtained, and confirmed that it was an objective product from the size that compared the marker by the 2% agarose gel electrophoresis, including the ethidium bromide. The project was completed with the help of Shanghai Sunny Biotechnology Co., Ltd. (Shanghai, China).

#### 2.2.3. Small Intestinal Immunohistochemistry

For all histological endpoints in small intestinal tissues, the samples were fixed in 10% neutral buffered formalin, embedded in paraffin, and sectioned at 5 μm. Tissue sections were dewaxed, rehydrated, and then stained. Lysozyme analysis of small intestinal sections was done by immunohistochemistry (HRP/DAB (ABC) IHC Detection Kit, Abcam, UK), following manufacturer recommendations. Anti-lysozyme antibody (ab108508) was purchased from Abcam, UK. Images were captured by microscopy (Olympus, Tokyo, Japan). The DAB staining intensity was analyzed using ImageJ (1.8.0_172) and represented by the average optical density (AOD) value (AOD = IOD/area, IOD: integrated option density). We examined five successive fields as a section, and three sections per mouse were used for analysis. The evaluation of staining intensity was performed by two independent assessors.

#### 2.2.4. Hematoxylin and Eosin (HE) Staining

For all histological endpoints in lung tissues, the tissues were fixed in 10% neutral buffered formalin, embedded in paraffin, and sectioned at 5 μm. Tissues were dewaxed, rehydrated, and stained. Morphological analysis of lung tissue sections was done by HE staining (Beyotime HE Staining Kit, Shanghai, China), following manufacturer recommendations. Histological changes were analyzed by a blinded participant using a 4-point scale as follows [18]: No detectable inflammation—0, bronchioles surrounded by a few inflammatory cells—1, bronchioles surrounded by a layer one cell deep—2, bronchioles surrounded by a layer 2–4 cells deep—3, bronchioles surrounded by a layer more than four cells deep—4. A minimum of 3–4 locations on each section (4 sections per slide), 3 slides, and *n* = 2–3 per group were used for analysis. All histology sections were imaged with bright field illumination at 20×. The evaluation of the Histological score was performed by two independent assessors.

#### 2.2.5. Myeloperoxidase (MPO) Assay

After the lung tissue was homogenized, activities of MPO in lung tissues were detected according to the instructions of the MPO Activity Colorimetric Assay Kit (BioVision Incorporated, Waltham, MA, USA). In BioVision’s MPO Assay Kit, HClO produced from H_2_O_2_ and Cl^−^ reacts with taurine to generate taurine chloramine, which subsequently reacts with the trinitrobenzene probe to eliminate the color (λ = 412 nm).

#### 2.2.6. Plasma Markers of Bacterial Translocation

Blood samples were collected from the mouse heart by cardiac puncture into an EDTA-rinsed syringe. Plasma was obtained by centrifuging the blood with 1500 g for 15 min at 4 °C. Samples were stored at −80 °C until use. Plasma lipopolysaccharide-binding protein (LBP) (Abcam, ab269542), sCD14 (RandD, Minneapolis, MN, USA, MC140) and Zonulin (MyBioSource, San Diego, CA, USA, MBS3806746) were quantified by ELISA according to the manufacturer’s instructions.

### 2.3. The 16S rRNA Gene Amplicon Sequencing and Bioinformatics

Feces and lung tissues of mice in the control group, sham group (at 3 h, 1 d, 3 d, 7 d and 14 d) and sTBI group (at 3 h, 1 d, 3 d, 7 d and 14 d) were collected in a sterile environment and stored in liquid nitrogen immediately. 16S ribosomal RNA (rRNA) sequencing was performed to classify bacterial DNA, which was isolated from the fecal and lung tissues microbiome. Quantitative polymerase chain reaction (PCR) of the bacterial 16S rRNA gene V3-V4 region was performed using the forward primer F (5′-ACTCCTACGGGAGGCAGCA-3′) and the reverse primer R (5′-GGACTACHVGGGTWTCTAAT-3′). The data from the 16S rRNA gene sequence were analyzed using QIIME 2 (2019.4) and R (v3.2.0) software packages. SourceTracker, popularly used in tracing the origin of bacteria, applies a Bayesian approach to determine the relative contributions of one or more designated “sources” to a particular “sink” microbiota. It also models the uncertainty regarding known and unknown source environments [19]. We employed SourceTracker to assess the contributions to the lung microbiota.

### 2.4. Statistical Analysis Methods

All experiments were repeated at least 6 times, which made the study reproducible. All data are expressed as mean ± SD. Comparisons between groups were tested by one-way analysis of variance (Tukey’s test). The Divisive Amplicon Denoising Algorithm 2 (DADA2) was used to dereplication and produce the amplicon sequence variants. Alpha (α) diversity was analyzed by the Kruskal-Wallis test. The difference between different groups was analyzed based on the principal coordinate analysis (PCoA) of the Bray-Curtis distance (Anosim). The distance to Q had a statistic difference based on permutational multivariate analysis of variance (PERMANOVA). Linear discriminant analysis (LDA) coupled with effect size measurements (LEfSe) was performed (http://huttenhower.sph.harvard.edu/galaxy/, accessed on 29 July 2020) to discover highly-dimensional lung bacteria and gut bacteria. Spearman rank correlation coefficient to analyze the correlation between intestinal apoptosis/antimicrobial peptides and gut dysbacteriosis/bacterial translocation. SPSS 23.0 software was used to complete the statistical test. *p* < 0.05 was considered significantly different.

## 3. Results

### 3.1. sTBI Alters the Lung Microbial Structure

**Figure 1:**
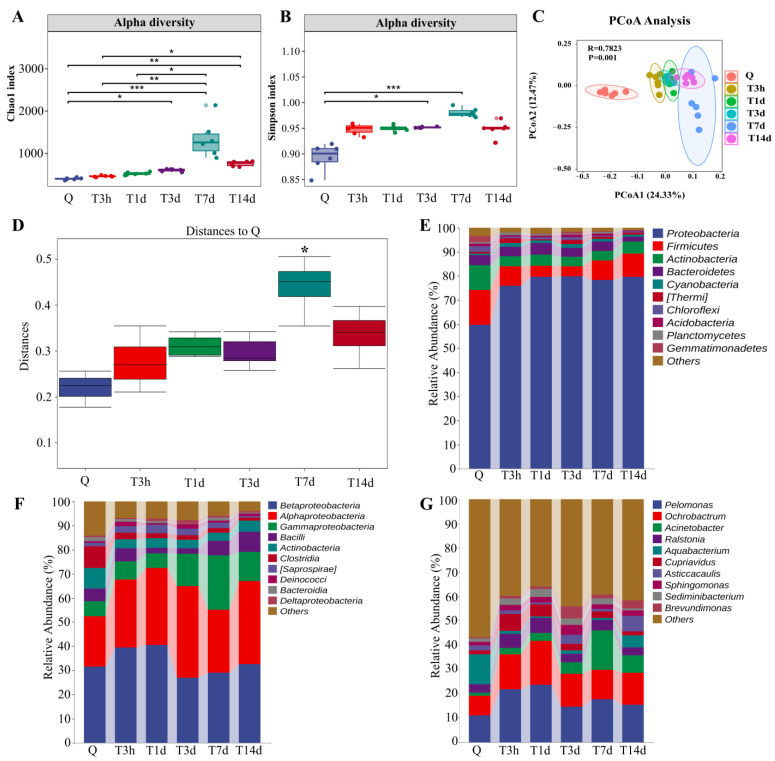
sTBI alters the lung microbial structure. (**A**,**B**): Analysis of alpha diversity of lung microbiota by Chao1 analysis and Simpson analysis, respectively. (**C**): PCoA plots of beta diversity in different groups. (**D**): Analysis of differences between groups based on permutational multivariate analysis of variance. (**E**–**G**): Relative abundance of lung microbiota at phylum, class and genus, respectively. Q: control group. T3h, T1d, T3d, T7d and T14d: 3 h, 1 d, 3 d, 7 d and 14 d after sTBI. * *p* < 0.05; ** *p* < 0.01; *** *p* < 0.001.

To explore the changes in lung microbiota after sTBI, we carried out 16S RNA sequencing on lung samples of mice from different groups or timepoints. The lung microbiota community in the sTBI group was significantly separated from the control group. The alpha diversity analysis was conducted to evaluate the richness and diversity of bacterial species. As illustrated in Figure 1A, the Chao1 index increased, peaking at 7 d after sTBI, which was significantly different from that of control mice (*p* < 0.05 with the Kruskal-Wallis test and post hoc Dunn’s test). At 3 d and 7 d after sTBI, the Simpson index was considered significantly higher than in control mice (*p* < 0.05 with Kruskal-Wallis test and post hoc Dunn’s test, Figure 1B). We also found that the Chao1 index and Simpson index did not differ statistically from control mice (*p* > 0.05). The data showed in Figure S1A. Beta diversity was assessed using Bray-Curtis similarity to represent between-sample similarities. Based on the Bray-Curtis distance, Principal Coordinate Analysis (PCoA) suggested that sTBI had significant effects on the lung microbiota structure (*R* = 0.78, *p* = 0.001, Anosim, Figure 1C). The distance to Q had a statistical difference at 7 d after sTBI (*p* = 0.016) based on PERMANOVA (Figure 1D). According to PERMANOVA, the distance to Q had no significant difference in the sham group (*p* > 0.05) (Figure S2D).

To further identify the critical bacteria caused by sTBI, we compared the relative abundances of microbes at various taxon levels among different groups or timepoints. At the phylum level, the top four bacteria found in the lungs of normal mice are *Proteobacteria* (59.72%), *Firmicutes* (14.49%), *Actinobacteria* (10.19%) and *Bacteroidetes* (4.11%) (Figure 1E). After sTBI, the relative abundance of *Proteobacteria* in the lung increased significantly. The proportions in control group, 3 h, 1 d, 3 d, 7 d and 14 d after sTBI were 59.72%, 75.89%, 79.57%, 79.76%, 78.35% and 79.70%. The lungs of normal mice were *betaproteobacteria* (31.56%), *alphaproteobacteria* (20.89%) and *gammaproteobacteria* (6.20%) at the class level (Figure 1F). *Gammaproteobacteria* levels increased significantly at 7 d after sTBI, reaching 22.39% (Figure 1F). At the genus level, the relative abundance of *Acinetobacter* in the lungs of control mice was only 1.37% and increased to 16.26% at 7 d after sTBI (Figure 1G). At different time points, the relative abundance of lung microbiota at phylum and genus levels remained relatively stable between the control group and sham group (Figure S1D,E).

**Figure 2:**
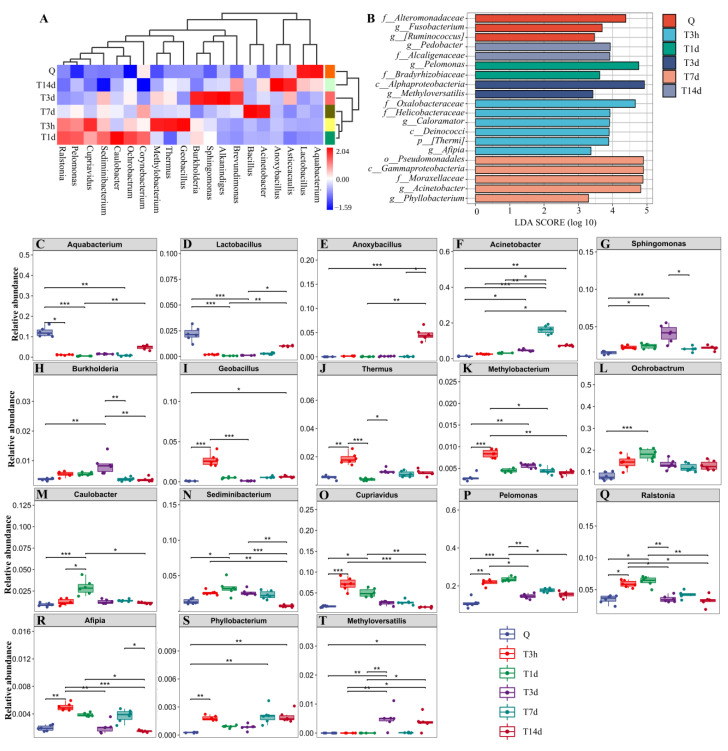
Species composition analysis of lung microbiota. (**A**): Heatmap analysis of relative abundances of gut microbiota at the genus level in different groups. (**B**): LEfSe analysis of lung microbiota. The histogram of the Linear discriminant analysis (LDA) scores illustrates the differentially abundant bacterial communities in the lung microbiota. The LDA score at log 10 > 3 is set as the threshold and the length of each bin, i.e., the LDA score represents the extent to which the bacterial biomarker differs among the groups. (**C**–**T**): Relative abundances of 18 significantly altered bacterial genera. Q: control group. T3h, T1d, T3d, T7d and T14d: 3 h, 1 d, 3 d, 7 d and 14 d after sTBI. * *p* < 0.05; ** *p* < 0.01; *** *p* < 0.001.

The heat map was used for species composition analysis to assess the variation in species composition between groups (or time points) and display the distribution trend of species abundance for each group (or time point). The results demonstrated that the lung bacteria of mice significantly deviated from normal at 7 days after sTBI. The bacteria in the control group were mainly *Aquabacterium* and *Lactobacillus*. However, the lung microbiota deviated from normal at 3 h after sTBI, and *Acinetobacter* and *Bacillus* dominated at 7 d after sTBI (Figure 2A). Furthermore, we evaluated a significantly abundant bacterial population using LefSe which was described as an important biomarker. The biomarkers were estimated using the LDA scores [(log10) > 3 as threshold] indicated by the histogram. At 7 d after sTBI, the prevalence of bacterial species belonging to *Acinetobacter* and *Phyllobacterium* were selected as biomarkers for lung microbiota (Figure 2B). As demonstrated in Figure 2C–T, the overall 18 genera significant differences between the control and sTBI group were detected.

The above findings show that sTBI causes lung microbiota imbalance in mice, whereas sham surgery, has no significant effect on lung microbiota structure.

### 3.2. sTBI Induces Gut Microbiota Dysbiosis

**Figure 3:**
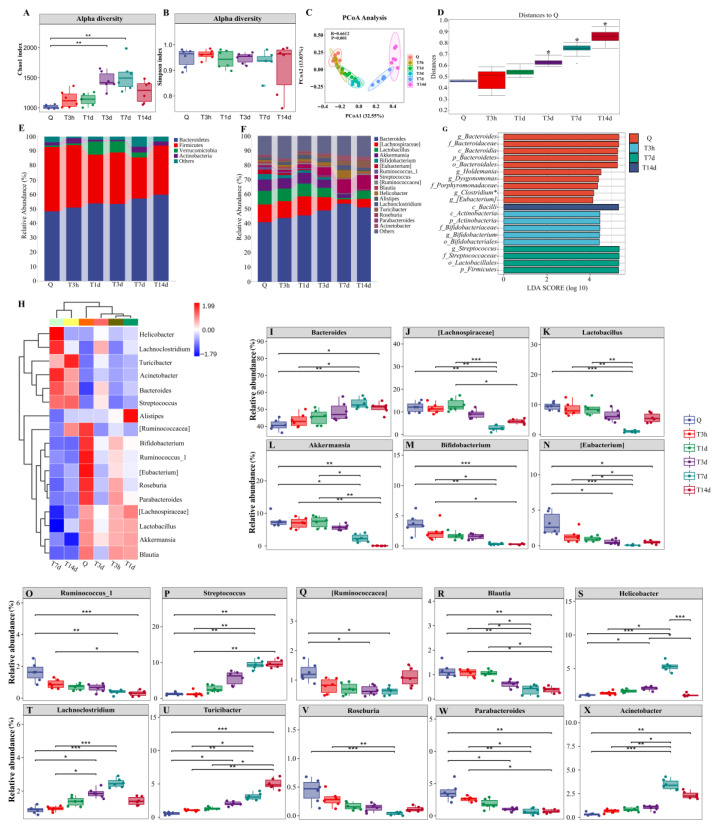
sTBI induces gut microbiota dysbiosis. (**A**,**B**): Analysis of alpha diversity of gut microbiota by Chao1 analysis and Simpson analysis, respectively. (**C**): PCoA plots of beta diversity in different groups. (**D**): Analysis of differences between groups based on permutational multivariate analysis of variance. (**E**,**F**): Relative abundance of gut microbiota at phylum and genus, respectively. (**G**): LEfSe analysis of gut microbiota. The histogram of the Linear discriminant analysis (LDA) scores illustrates the differentially abundant bacterial communities in the gut microbiota. The LDA score at log 10 > 3 is set as the threshold and the length of each bin, i.e., the LDA score represents the extent to which the bacterial biomarker differs among the groups. (**H**): Heatmap analysis of relative abundances of gut microbiota at the genus level in different groups. (**I**–**X**): Relative abundances of 16 significantly altered bacterial genera. Q: control group. T3h, T1d, T3d, T7d and T14d: 3 h, 1 d, 3 d, 7 d and 14 d after sTBI. * *p* < 0.05; ** *p* < 0.01; *** *p* < 0.001.

As illustrated in Figure 3A, the Chao1 index increased after sTBI and peaked at 7 d (*p* < 0.05 with the Kruskal-Wallis test and post hoc Dunn’s test). The Simpson index was not significantly different from control mice (*p* > 0.05) (Figure 3B). Based on the Bray-Curtis distance, PCoA demonstrated that sTBI had a significant influence on the gut microbiota structure (*R* = 0.66, *p* = 0.001, Anosim, Figure 3C). The distance to Q had a statistic difference at 3 d, 7 d and 14 d after sTBI (*p* < 0.05) based on Permutational multivariate analysis of variance (PERMANOVA) (Figure 3D). To further identify the critical bacteria caused by sTBI, we compared the relative abundances of microbes at various taxon levels among different groups or time points. In normal mice, *Bacteroidetes* (48.28%) and *Firmicutes* (44.53%) were the dominant phyla (Figure 3E). After 7 d of sTBI, the relative abundance of *Bacteroidetes* in the gut increased to 57.08%, while *Firmicutes* decreased to 28.50% (Figure 3E). Figure 3F shows the top 17 bacteria at the genus level. Following sTBI, the relative abundance of *[Lachnospiraceae]*, *Lactobacillus*, *Akkermansia*, *Bifidobacterium*, *Roseburia*, ect. in the gut decreased, whereas *Bacteroides*, *Streptococcus*, *Lachnoclostridium*, *Turicibacter*, *Acinetobacter*, etc. increased. More results are shown in Figure 3F.

In the sham group, the Chao1 index and Simpson index in the sham group were not significantly different from control mice (*p* > 0.05). The data showed in Figure S2A,B. As per PERMANOVA, the distance to Q had no statistical difference in the sham group (*p* > 0.05) (Figure S2C). At different time intervals, the relative abundance of gut microbiota at phylum and genus levels remained relatively stable between the control group and sham group (Figure S2D,E).

Furthermore, we evaluated a significantly abundant bacterial population using LefSe which was described as an important biomarker. The biomarkers were estimated using the LDA scores [(log10) > 3 as threshold] indicated by the histogram (Figure 3G). The prevalence of *[Eubacterium]*, *Bifidobacterium* and *Streptococcus* bacterial species were identified as biomarkers for gut microbiota at Q, 3 h and 7 d after sTBI, respectively (Figure 3G). A heat map was used for species composition analysis. In the control group, the bacteria were predominantly *[Ruminococcacea]*, *Bifidobacterium*, *Ruminococcus_1*, *[Eubacterium]*, *Roseburia*, *Parabacteroides*, *[Lachnospiraceae]*, *Lactobacillus*, *Akkermansia* and *Blautia* (Figure 3H). Three hours after sTBI, the above bacteria were reduced, whereas conditional pathogens such as *Lachnoclostridium*, *Acinetobacter*, *Bacteroides* and *Streptococcus* grew significantly (Figure 3H). As demonstrated in Figure 3I–X, the overall 16 genera significant differences between the control and sTBI group were detected.

The results confirmed that the gut microbiota of sTBI mice was significantly altered and the bacterial biomarkers were identified.

### 3.3. Lung Injury and Bacterial Translocation in Mice after sTBI

**Figure 4:**
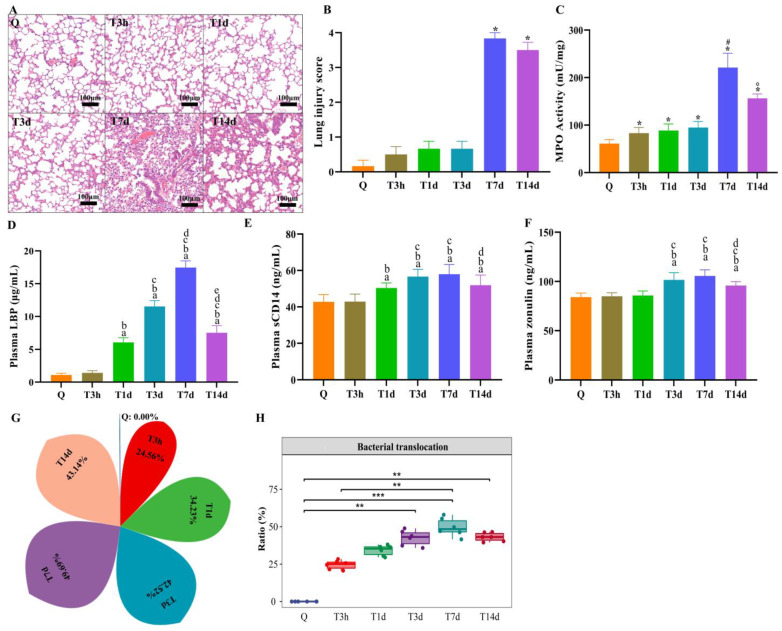
Lung injury and bacterial translocation and in mice after sTBI. (**A**): Lung HE staining. Q: control group. T3h, T1d, T3d, T7d and T14d: 3 h, 1 d, 3 d, 7 d and 14 d after sTBI. Scale bar = 100 μm. (**B**): Quantification of histological injury score. Data are depicted as mean ± SD with * *p* < 0.05 compared to Q, T3h, T1d, T3d by one-way ANOVA. (**C**): MPO activity of lung tissue. Q: control group. Data are depicted as mean ± SD with * *p* < 0.05 compared to Q, ^#^*p* < 0.05 compared to Q, T3h, T1d, T3d, ° *p* < 0.05 compared to T7d by one-way ANOVA. (**D**): Levels of plasma LBP. ^a^ *p* < 0.05 compared to Q, ^b^ *p* < 0.05 compared to T3h, ^c^ *p* < 0.05 compared to T1d, ^d^ *p* < 0.05 compared to T3d, ^e^ *p* < 0.05 compared to T7d by one-way ANOVA. (**E**): Levels of plasma sCD14. ^a^ *p* < 0.05 compared to Q, ^b^ *p* < 0.05 compared to T3h, ^c^ *p* < 0.05 compared to T1d, ^d^ *p* < 0.05 compared to T7d by one-way ANOVA. (**F**): Levels of plasma zonulin. ^a^ *p* < 0.05 compared to Q, ^b^ *p* < 0.05 compared to T3h, ^c^ *p* < 0.05 compared to T1d, ^d^ *p* < 0.05 compared to T7d by one-way ANOVA. Q: control group. T3h, T1d, T3d, T7d and T14d: 3 h, 1 d, 3 d, 7 d and 14 d after sTBI. Data are depicted as mean ± SD. (**G**,**H**): SourceTracker identified lung microbiota contains bacteria from the gut microbiota. * *p* < 0.05; ** *p* < 0.01; *** *p* < 0.001.

Lung damage was analyzed by HE staining and MPO activity. There was significant peribronchial inflammation surrounding the bronchioles in mice exposed to sTBI at 7 d and 14 d compared to the controls (Figure 4A). At 7 d and 14 d, the histology scores for sTBI were significantly higher than the control group (*p* < 0.05) (Figure 4B). The MPO activity, which was increased at all-time points after sTBI compared to control mice and peaked at 7 d (*p* < 0.05), was used to assess the appearance and activation of neutrophils in lung tissue (Figure 4C). The histology scores for the sham group at 7 d were significantly higher than the control group (*p* < 0.05) (Figure S3A,B). MPO activity in the sham group increased at 7 d compared to control mice (*p* < 0.05) (Figure S3C).

To further assess whether the bacteria spread through the bloodstream, we examined biomarkers of bacterial translocation in plasma-like as LBP, sCD14 and Zonulin. The LBP level increased significantly, peaking at 7 d after sTBI (*p* < 0.05) (Figure 4D). Another biomarker of bacterial translocation in plasma is sCD14. The results showed that sCD14 increased significantly after sTBI and peaked at 7 d (*p* < 0.05) (Figure 4E). Zonulin has been shown to correlate with gut permeability and be used to assess impaired gut barrier function. It was found that the Zonulin increased significantly at 3 d after sTBI and maintained the high level (*p* < 0.05) (Figure 4F). There were no significant differences in translocation biomarkers in plasma, such as LBP, sCD14, and Zonulin, between the sham group and the control group at any point (*p* > 0.05) (Figure S3D–F). The findings suggested that sTBI could cause gut barrier disruption and bacterial translocation via blood.

We further used SourceTracker to determine the relative contribution of the gut microbiota to the lung after sTBI. The lung microbiota represents 0% gut source of control and sham mice, 24.56% gut source at 3 h after sTBI, and 49.69% gut source at 7 d after sTBI, as per SourceTracker (Figure 4G,H). The findings confirmed that sTBI induced translocation of gut microbiota to the lungs.

### 3.4. Paneth Cells Control of Gut Microbiota and Post-sTBI Infection

**Figure 5:**
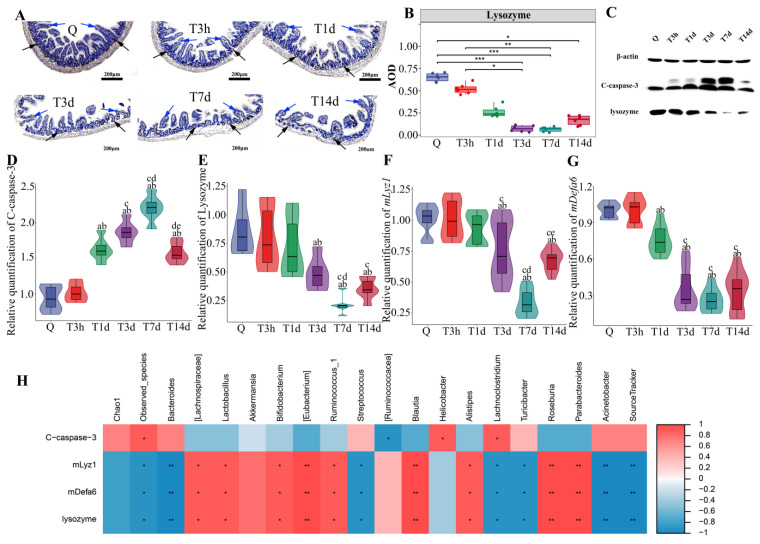
Paneth cells control gut microbiota and post-sTBI infection. (**A**): Intestinal lysozyme immunohistochemistry. Q: control group. T3h, T1d, T3d, T7d and T14d: 3 h, 1 d, 3 d, 7 d and 14 d after severe TBI. The black arrow indicated lysozyme. The Blue arrow indicates the villi of the small intestine. Scale bar = 200 μm. (**B**): The DAB staining intensity. * *p* < 0.05; ** *p* < 0.01; *** *p* < 0.001. (**C**): Apoptosis and lysozyme expression in the small intestine by Western Blot. C-caspase-3 indicated Cleaved caspase-3. (**D**): Cleaved caspase-3 at different time points after severe TBI. Data are depicted as mean ± SD with ^a^ *p* < 0.05 compared to Q, ^b^ *p* < 0.05 compared to T3h, ^c^ *p* < 0.05 compared to T1d, ^d^ *p* < 0.05 compared to T3d, ^e^ *p* < 0.05 compared to T7d by one-way ANOVA. (**E**): Lysozyme at different time points after severe TBI. Data are depicted as mean ± SD with ^a^ *p* < 0.05 compared to Q, ^b^ *p* < 0.05 compared to T3h, ^c^ *p* < 0.05 compared to T1d, ^d^ *p* < 0.05 compared to T3d by one-way ANOVA. (**F**): *mLyz1* at different time points after severe TBI. Data are depicted as mean ± SD with ^a^ *p* < 0.05 compared to Q, ^b^ *p* < 0.05 compared to T3h, ^c^ *p* < 0.05 compared to T1d, ^d^ *p* < 0.05 compared to T3d, ^e^ *p* < 0.05 compared to T7d by one-way ANOVA. (**G**): *mDefa6* at different time points after severe TBI. Data are depicted as mean ± SD with ^a^ *p* < 0.05 compared to Q, ^b^ *p* < 0.05 compared to T3h, ^c^ *p* < 0.05 compared to T1d by one-way ANOVA. (**H**): The correlation between intestinal apoptosis or antimicrobial peptides and gut dysbacteriosis or bacterial translocation by Spearman rank correlation coefficient. Red indicated a positive correlation. Blue indicated a negative correlation. Color depth indicates the strength of correlation. * *p* < 0.05; ** *p* < 0.01; *** *p* < 0.001.

Lysozyme is primarily expressed in Paneth cells of the small intestine. Immunohistochemical staining showed a decrease of lysozyme (Black arrow) at 3 days after sTBI (Figure 5A). Moreover, immunohistochemistry analysis showed villous atrophy (Blue arrow) on days 1, 3, 7 and 14 after sTBI (Figure 5A). Semi-quantitative analysis of immunohistochemical results was conducted by Image J, and the results showed that lysozyme decreased to its lowest level 7 days after sTBI (*p* < 0.05) (Figure 5B). The sham group showed no villous atrophy as indicated in Figure S3G,H. The Western blot analysis revealed the presence of C-caspase-3 and lysozyme. The C-caspase-3 levels increased on the first day after sTBI, and peaked on day 7, and dropped, but remained elevated (*p* < 0.05) (Figure 5C,D). Furthermore, Western blot analysis revealed that sTBI significantly decreased lysozyme levels in Paneth cells 3 d after sTBI (*p* < 0.05), in consistence with the results of the immunohistochemistry analysis (Figure 5C,E). In addition, RT-PCR showed that *mLyz1* and *mDefa6* were decreased in Paneth cells (*p* < 0.05) (Figure 5F,G).

Therefore, we examined the relationship between intestinal apoptosis or antimicrobial peptides and gut dysbacteriosis or bacterial translocation using the Spearman rank correlation coefficient. Bacterial translocation was found to be negatively correlated with the expression level of antimicrobial peptides (*mlyz1*, *mdefa6* and lysozyme) in Paneth cells (*p* < 0.01) (Figure 5H). Gut beneficial bacteria (*[Lachnospiraceae]* *, *Lactobacillus* *, *Bifidobacterium* *, *Ruminococcus_1* *, *Alistipes* *, *[Eubacterium]* **, *Blautia* **, *Roseburia* **, *Parabacteroides* **) correlated positively with antimicrobial peptides expression (*mlyz1*, *mdefa6* and lysozyme) in Paneth cells (*p < 0.05, ** *p* < 0.01) (Figure 5H). There was a negative correlation between gut conditional pathogens (*Streptococcus* *, *Lachnoclostridium* *, *Turicibacter* *, *Bacteroides* **, *Acinetobacter* **) and antimicrobial peptides expression (*mlyz1*, *mdefa6* and lysozyme) in Paneth cells (*p < 0.05, **p < 0.01) (Figure 5H).

## 4. Discussion

Brain-gut axis (BGA) is a complex network of bidirectional communication between the gut, its microbiome, and the nervous system [20]. The gut microbiome is well known to have a pivotal role in the regulation of the health and behavior of the host, affecting digestion, metabolism, immunity, as well as changes in bones, muscles and the brain [21]. The TBI is reported to have destroyed gut mucosal integrity, and the leaky gut may allow bacterial components to enter circulation [3,22,23]. Overall rates of lower respiratory tract infections in patients with sTBI have been reported to be as high as 24% to 72% [24,25]. Recent studies have shown that the lung microbiota of patients with sepsis, acute res-piratory distress syndrome and stroke are often found to be rich in gut-related bacteria [6,7,8,9]. Our data indicate that gut bacteria translocated to the lungs after sTBI, and Paneth cells may regulate gut microbiota stability and translocation.

Previously, healthy lungs were considered sterile on the basis of classical culture studies. However, the existence of a healthy lung microbiota which includes *Firmicutes*, *Bacteroidetes*, *Proteobacteria* and *Actinobacteria,* is now widely accepted [26,27]. Alpha diversity is characterized by the total number of species (species richness), the relative abundances of the species (species evenness), or indices that combine these two dimensions [28]. The microbial diversity decreases with disease progression, and lung disease is associated with lower microbial diversity [29,30]. However, some studies hold the opposite view. It was reported that smoking increased lung microbial diversity, implying that smoke exposure increased the risk of bacterial infection, thereby increasing microbial diversity [31]. Another study found that non-tumor adjacent or tumor tissue had higher alpha diversity than normal lung [32]. Our findings showed that the alpha diversity of the lung microbiota was significantly higher in sTBI mice than in control mice. Hence, the evaluated richness and diversity of the lung microbiome may suggest an imbalanced lung milieu. The extent of the similarity between microbial communities based on the degree of structural overlap was characterized by beta diversity (inter-sample diversity) [33]. The repeatability of samples in each group was determined to be good based on PCoA analysis, and the difference between lung samples was significant.

The phyla *Proteobacteria*, *Firmicutes*, *Bacteroidetes* and *Actinobacteria* were found to dominate the lung microbiome in C57BL/6 mice at all the stages of development [34]. In our study, *Proteobacteria*, *Firmicutes*, *Actinobacteria* and *Bacteroidetes* were the top four relative abundances of bacteria in the lungs of control mice. We found that the lungs of control mice were rich in *Lactobacillus*, which was significantly reduced after sTBI, consistent with others [35]. *Lactobacillus* is a kind of probiotic, which has the functions of anti-inflammatory, anti-infection and regulating immune function, as a therapeutic approach to the prevention of lung diseases like asthma and allergic reactions, viral infections and chronic obstructive pulmonary disease [36]. We found that *Bacillus* and *Acinetobacter* in the lungs were significantly increased at 7 d after sTBI. *Bacillus* is still the largest genus, and it continues to accommodate most of the best-known names like *Bacillus anthracis*, *Bacillus cereus*, *Bacillus licheniformis*, *Bacillus subtilis* and *Bacillus pumilus*. The bacteria have been reported from a wide range of opportunistic infections both in immunocompromised and in immunocompetent patients or those with implanted devices or trauma [37]. The genus *Acinetobacter* comprises a complex and heterogeneous group of bacteria, many of which can cause a range of opportunistic, often catheter-related infections in humans [38]. *Acinetobacter* infections have spread rapidly through hospitals globally, and the two most common clinical manifestations of *Acinetobacter baumannii* are nosocomial pneumonia and bacteremia [39]. Similarly, alpha and beta diversity of gut microbiota in sTBI mice were significantly altered. The findings showed that sTBI induced gut dysbacteriosis with decreased beneficial bacteria and increased harmful bacteria.

Perturbed gut integrity and permeability (“leaky gut”) may result in microbial (bacterial) translocation, and the eventual leakage of bacteria or their metabolites into the circulation, which can make the host susceptible to various types of diseases via inducing chronic or acute inflammatory response [40]. The LBP, sCD14 and Zonulin are biomarkers of microbial translocation [41,42,43]. It was discovered that Zonulin correlates with gut permeability and be used as a measurement of impaired gut barrier function. Our findings demonstrated that gut barrier disruption and bacterial translocation via blood after sTBI. The SourceTracker algorithm was used to reveal the possible origins of microbial communities that seeded the lungs in post-stroke mice [8]. It was reported that more than 60% of the bacterial communities present in the lung microbiota were predicted to originate from the digestive system [8]. In our study, SourceTracker identified that the lung tissue microbiota reflects 49.69% gut source at 7 days after sTBI. The findings confirmed that sTBI caused the translocation of gut microbiota to the lungs.

In patients with brain injury, gastrointestinal dysfunction is a common problem [10]. The intestinal mucosa is the largest surface of interaction between the internal milieu and the external environment, preventing the invasion of pathogenic antigens [44]. After brain injury, intestinal epithelial cell dysfunction or apoptosis may occur because of ischemia and/or hypoxia, oxidative stress, and inflammatory reaction [11]. TBI is associated with disruption of the hypothalamic–pituitary–adrenal axis and autonomic nervous system, leading to the abnormal secretion of glucocorticoids and catecholamines, respectively [45]. The excessive production of neurotransmitters can reduce gut motility and mucosa integrity [45]. Moreover, TBI leads to an acute inflammatory response in the intestine involving the production of inflammatory cytokines have been noted to cause mucosal destruction in rat models. A detailed review on the topic has been published [46]. The mechanisms are briefly described as follows. First, the tight junction proteins were disrupted by inflammatory cytokines after TBI. Second, inflammatory cytokines such as interleukin type 6 and tumor necrosis factor α result in apoptosis of the intestinal epithelial cells. One of the mechanisms that could lead to intestinal damage is oxidative stress. TBI can cause intestinal mucosal ischemia or hypoperfusion, and lead to intestinal injury through oxidative stress. It was reported that annexin A5 inhibits TBI-induced intestinal injury by restraining oxidative stress and inflammatory responses [47]. TBI-induced intestinal injury can cause the translocation of endotoxins and bacteria in the intestinal tract, further inducing or aggravating the systemic inflammatory response and resulting in multiple organ failures and death [48].

Paneth cells are the primary cell producing antimicrobial peptides in the small intestine and play an important role in maintaining the steady-state of intestinal microbiota [49]. Moreover, Paneth cells are professional secretory cells that control enteric bacteria by secreting antimicrobial substances into the lumen, and antimicrobial proteins, α-defensins, are key contributors to microbiota regulation [50,51]. We analyzed the expression of lysozyme, *mlyz1* and *mdefa6* by Western Blot and RT-PCR, implying that small intestinal antimicrobial peptides decreased significantly. The mechanisms of intestinal injury caused by TBI may be the factors of Paneth cell dysfunction, which requires further investigation. Furthermore, we examined the correlation between pathological data of the small intestine and dysbacteriosis using the Spearman rank correlation coefficient. The degree of bacterial translocation was negatively correlated with the expression of antimicrobial peptides. There was a positive correlation between gut-beneficial bacteria and the expression level of antimicrobial peptides. There was a negative correlation between gut conditional pathogens and the expression level of antimicrobial peptides. The findings imply that Paneth cell antimicrobial peptides may affect the stability and translocation of gut microbiota.

## 5. Conclusions

Our findings showed that sTBI-induced gut dysbacteriosis with decreased beneficial bacteria and increased pathogenic bacteria. After sTBI, the lung microbiota contains gut taxa, according to SourceTracker, a popular tool for tracing the origin of bacteria. In the small intestine, sTBI caused gastrointestinal dysfunction by increasing apoptosis and decreasing antimicrobial peptides. Paneth cell antimicrobial peptides may influence microbiota stability and transfer in the gut.

## Data Availability

Raw sequences obtained in this study were submitted to NCBI Sequence Read Archive database under the accession number of PRJNA774905.

## Supplementary Materials

The following supporting information can be downloaded at: https://www.mdpi.com/article/10.3390/microorganisms10102082/s1, Figure S1: Relatively stable of lung microbiota in sham group; Figure S2: Relatively stable of gut microbiota in sham group; Figure S3: Relatively stable of histopathology and biomarkers of bacterial translocation in plasma.
