# Supplementary material for: Translocation and Dissemination of Gut Bacteria after Severe Traumatic Brain Injury
Source: Microorganisms. 2022 Oct 21;10(10):2082. doi: 10.3390/microorganisms10102082 (PMC9611479; doi:10.3390/microorganisms10102082)
Supplement: Supplementary file 1 [file microorganisms-10-02082-s001.zip › Supplementary Figure legends.pdf]

Figure legends

Figure S1. Relatively stable of lung microbiota in sham group. **A-B**: Analysis of alpha diversity of lung microbiota by Chao1 analysis and Simpson analysis, respectively. **C**: Analysis of differences between groups based on permutational multivariate analysis of variance. **D-E:** Relative abundance of lung microbiota at phylum and genus, respectively. Q: control group. S3h, S1d, S3d, S7d and S14d: 3 h, 1 d, 3 d, 7 d and 14 d after sham surgery.

Figure S2. Relatively stable of gut microbiota in sham group. **A-B**: Analysis of alpha diversity of gut microbiota by Chao1 analysis and Simpson analysis, respectively. **C**: Analysis of differences between groups based on permutational multivariate analysis of variance. **D-E:** Relative abundance of gut microbiota at phylum and genus, respectively. Q: control group. S3h, S1d, S3d, S7d and S14d: 3 h, 1 d, 3 d, 7 d and 14 d after sham surgery.

Figure S3. Relatively stable of histopathology and biomarkers of bacterial translocation in plasma. **A:** Lung HE staining. **B:** Quantification of histological injury score. Data are depicted as mean ± SD with $^{*}P < 0.05$ compared to Q, $^{\#}P < 0.05$ compared to S3h by one-way ANOVA. **C:** MPO activity of lung tissue. Q: control group. Data are depicted as mean ± SD with $^{*}P < 0.05$ compared to Q, $^{\#}P < 0.05$ compared to S3h by one-way ANOVA. **D-F:** Levels of plasma LBP, sCD14 and zonulin, respectively. **G:** Intestinal lysozyme immunohistochemistry. Black arrow indicated lysozyme. Blue arrow indicated the villi of the small intestine. Scale bar = 200μm. **H:** The DAB staining intensity. Q: control group. S3h, S1d, S3d, S7d and S14d: 3 h, 1 d, 3 d, 7 d and 14 d after sham surgery. Scale bar = 100μm.
