# Supplementary figures and images for: Translocation and Dissemination of Gut Bacteria after Severe Traumatic Brain Injury
Source: Microorganisms. 2022 Oct 21;10(10):2082. doi: 10.3390/microorganisms10102082 (PMC9611479; doi:10.3390/microorganisms10102082)

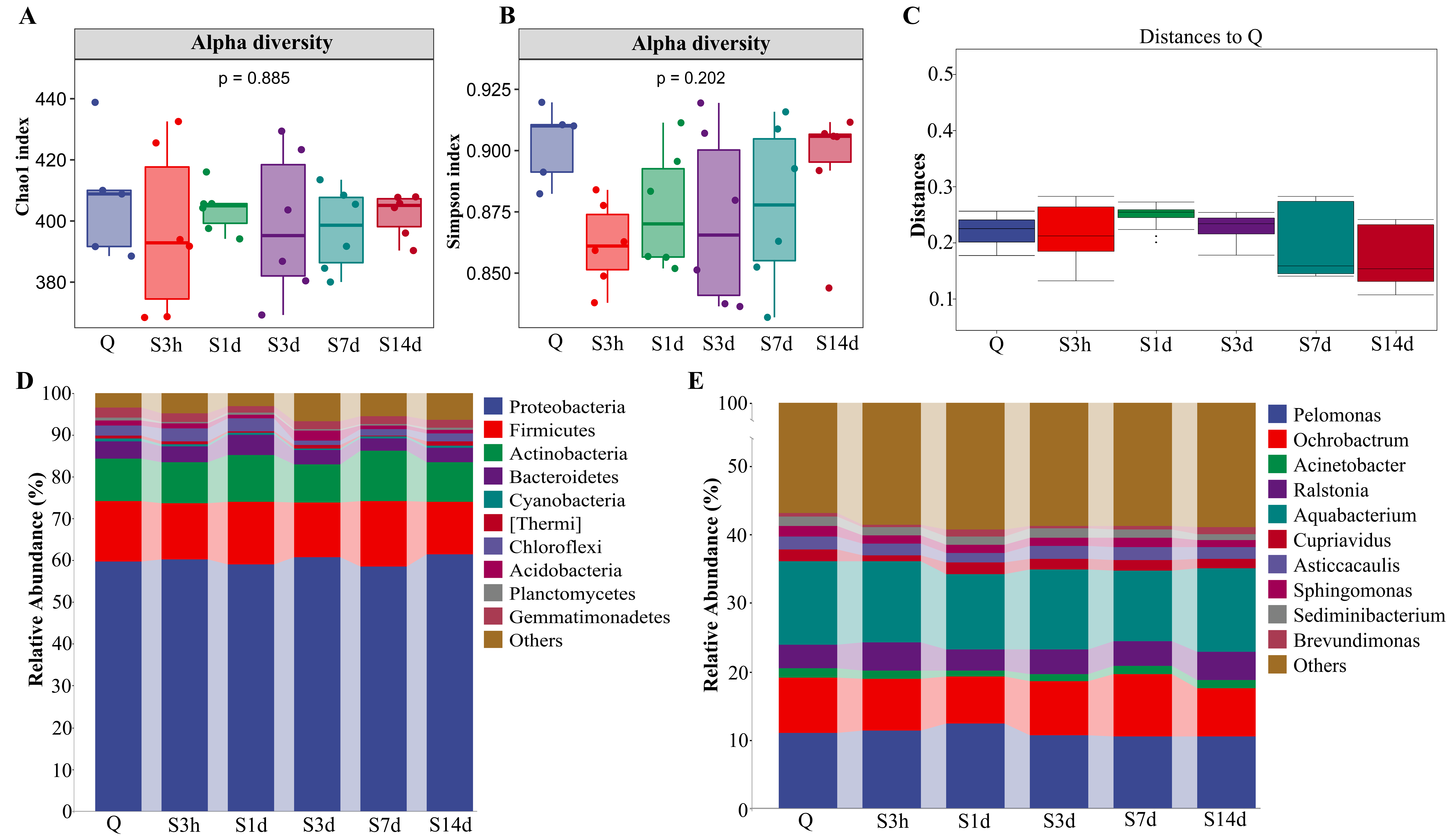

Supplement: Supplementary file 1 [file microorganisms-10-02082-s001.zip › Figure S1.tif]

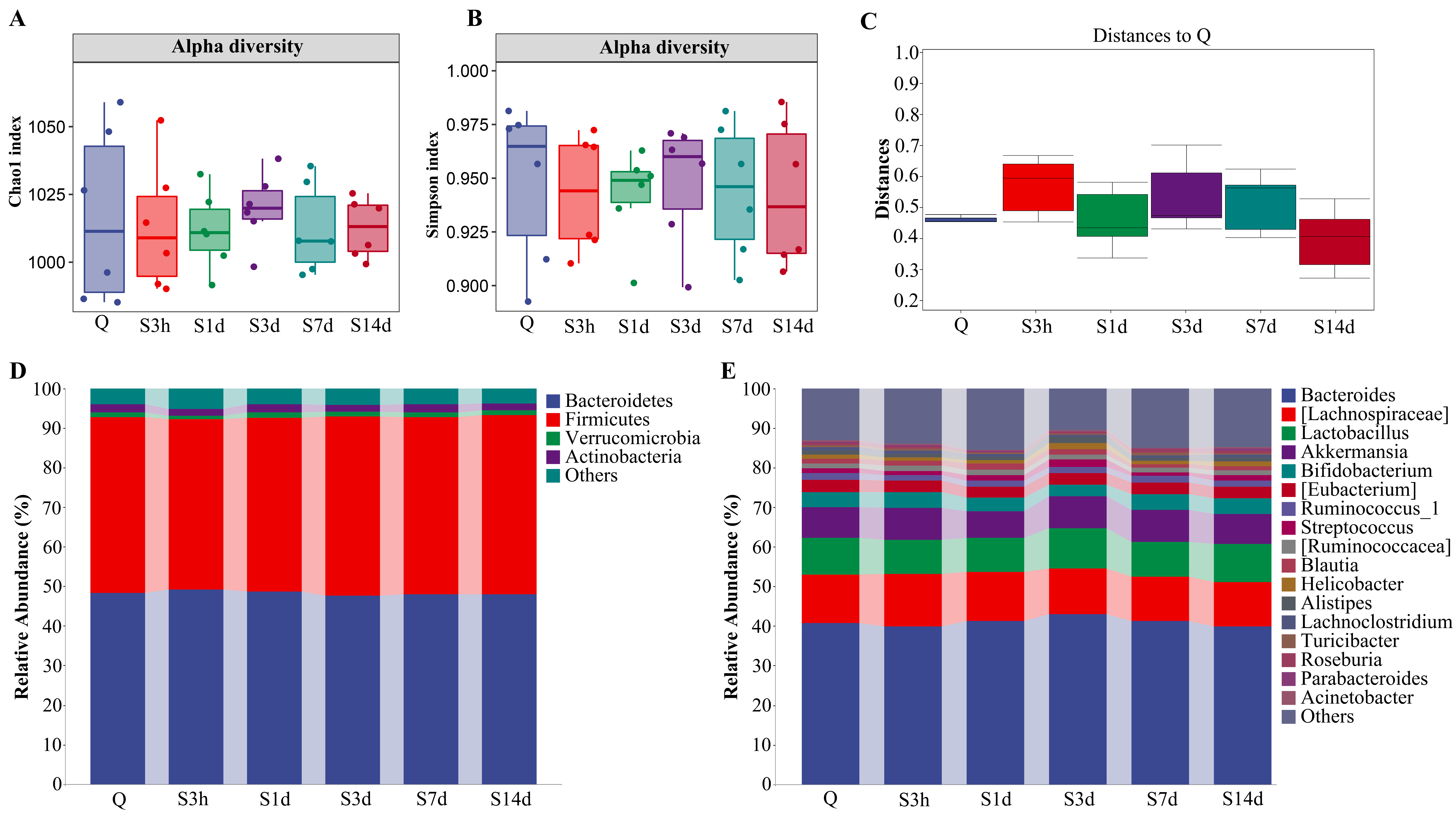

Supplement: Supplementary file 1 [file microorganisms-10-02082-s001.zip › Figure S2.tif]
